# Effect of Drying Methods on the Leaf and Flower Tissues of *Paulownia elongata* and *P. fortunei* and Resultant Antioxidant Capacity

**DOI:** 10.3390/antiox14030280

**Published:** 2025-02-27

**Authors:** Lubana Shahin, Ajit K. Mahapatra, Nirmal Joshee

**Affiliations:** 1 Agricultural Research Station, College of Agriculture, Family Sciences, and Technology, Fort Valley State University, Fort Valley, GA 31030, USA; ls32787@uga.edu (L.S.); mahapatraa@fvsu.edu (A.K.M.); 2 Complex Carbohydrate Research Center, The University of Georgia, Athens, GA 30605, USA

**Keywords:** antioxidant activity, color, drying, moisture content, Paulownia, tea

## Abstract

*Paulownia* leaves and flowers have been used to prepare medicinal tea in traditional Chinese medicine; however, there has been no scientific validation of bioactive compounds so far. A systematic study is presented to establish a suitable drying protocol for leaf and flower tissues that may be useful in preserving bioactive compounds and retaining high antioxidant capacity. Additionally, a suitable drying protocol is commercially imperative for improving the shelf life of these tissues. In this study, *P. elongata* and *P. fortunei* juvenile leaves and flowers at two stages (pre- and post-anthesis stages) were subjected to five drying treatments to study the drying characteristics and were analyzed for total polyphenols, total flavonoids, and antioxidant capacity. Oven drying, sun drying, shade drying, freeze drying, and microwave drying were the five drying methods that were used to evaluate their efficacy on the drying characteristics and antioxidant potential. Fresh and dried tissues were analyzed for total polyphenols, total flavonoids, total tannins, total catechins, total monomeric anthocyanins, and total antioxidant capacity. A strong correlation was observed between the moisture content and total polyphenols (the lower the moisture content, the lower the polyphenols). Sun drying was the best method for *Paulownia* tissues based on color retention, moisture content, overall cost effectiveness, time, and antioxidant capacity. Shade drying was the second-best method based on the same parameters. No significant differences were observed between *P. elongata* and *P. fortunei* tissues in their total antioxidant capacity. Leaves and flowers at the pre-anthesis stage (stage 6) registered a higher level of total polyphenols, flavonoids, tannins, catechins, and resultant antioxidant capacity in comparison with flowers at stage 9 (the post-anthesis stage). Monomeric anthocyanins were highest in *P. elongata* flowers at stage 6. A strong correlation was observed between moisture content and the antioxidant levels of *Paulownia* tissues.

## 1. Introduction

*Paulownia* (family *Paulowniaceae*) is a fast-growing tree represented by nine species and several natural hybrids [1,2]. *Paulownia* species are used in traditional Chinese medicine to treat various diseases, especially the ones related to lung dysfunction [3]. Various plant parts of *Paulownia* such as leaves, flowers, fruits, bark, wood, root, and seeds are rich in phenolic substances [4]. *Paulownia* has been used in China to make herbal tea, using leaves, flowers, and fruits (outer tissue of the fruit), individually or in combination [5]. Due to the reported health benefits, herbal tea has become more acceptable than caffeinated beverages [6]. Further, consuming herbal tea may be the easiest way to introduce bioactive compounds to the human body. The essential oils present in *Paulownia* flower impart a flavor to *Paulownia* tea [7]. *Paulownia* is rich in polyphenolic compounds and exhibits high antioxidant activity [3,8]. Phytochemical research conducted on Paulownia flowers reveals a complex chemical composition, full of bioactive compounds. These compounds fall into various groups but are mostly dominated by flavonoids, phenylpropanoids, terpenoids, volatiles, polysaccharides, lignans, and iridoids. A recent study has reported the phytochemical, antibacterial, and anticancer potential of various Paulownia species flowers including *P. fortunei*, *P. elongata*, *P. tomentosa*, and *P. australis* [9]. In a biological system, these compounds express antioxidant, anti-inflammatory, antibacterial, antiviral, anticancer, hypoglycemic, hypolipidemic, neuroprotective, and immunoregulation activities [9,10]. Various economic activities that can be initiated around Paulownia tree have been covered in the FVSU extension bulletin (https://www.fvsu.edu/content/userfiles/files/2023/02/paulownia-honey-brochure-WEB-1_0(1).pdf, accessed on 25 February 2025). Drying is used for preservation and increasing the shelf life of various food and medicinal plants [11]. Drying promotes the removal of water, which, in turn, slows down microbial spoilage and the deterioration of the product, enhancing shelf life [12,13]. Moreover, drying methods can influence the composition of bioactive compounds in medicinal plants [14]. Structural, chemical, and phytochemical changes occur in the final product because of drying that can affect sensory properties (texture and color) and nutritional values [15]. Among the various drying techniques, shade, freeze, microwave, hot air (oven), and sun drying are the most studied methods. Shade, sun, and hot air drying are the most used methods because of their lower cost and efficiency [13]. Shade drying provides products with an extended shelf life of up to a year [16]. Freeze drying prevents the deterioration and microbiological reactions, resulting in higher-quality final products with superior flavor and aroma and nutrition retention [13,17]. Microwave drying is popular for a variety of food products as it requires lesser time, yielding a final product of improved quality [18]. The type of drying treatment during the processing of fresh plant tissues directly affects the final composition of phytochemicals in tea and medicinal herbs and, hence, the quality [19]. It is imperative to determine the most suitable drying treatment that provides the end user a tea with an extended shelf life, desirable sensory properties, and health benefits.

Eight phenolic compounds with high bioactivity have been reported from *P. tomentosa* bark [20] and a high amount of catechin and β-carotene was extracted from the leaves and flowers [21]. To the best of our knowledge, no reports are available on the effect of various drying treatments on the drying time, final moisture content, water activity, color, total polyphenols, total flavonoids, total catechins, total tannins, total monomeric anthocyanins, and total antioxidant activity of *P. elongata* and *P. fortunei* leaves and flowers.

Phenolic compounds are ubiquitous across the plant kingdom and many of them possess bioactive properties related to a reduction in oxidative damage [22]. Polyphenols present in plants are a subject of great interest due to their effects on human health [23,24,25]. Monomeric anthocyanins are the free form of anthocyanins, cyanidin-3-glucoside being the most abundant one in plants [26]. Catechins are monomeric flavanols, and have anticancer, anti-obesity, anti-diabetic, anti-cardiovascular, anti-infectious, hepatoprotective, and neuroprotective properties [27], with tea being its richest source. Dietary sources that are rich in polyphenol are fruits, vegetables, and beverages like tea and coffee [24,28].

Bioactive ursolic acid, paulownin, and syringin have been extracted from the leaves, xylem, and bark tissues of *Paulownia*, respectively [20]. Apigenin, a flavonoid with anti-inflammatory, anti-spasmodic, antioxidant, and anti-tumorigenic properties, was reported in *P. tomentosa* flower extracts [29]. Out of 23 flavonoids isolated from *Paulownia tomentosa* fruits, five exhibited anti-inflammatory activity [30]. The *P. tomentosa* leaf extracts showed antiradical and cell protective effects due to their high flavonoid content [31]. In addition, compounds extracted from *Paulownia* tissues have also shown antibacterial, anti-inflammatory, thirst-quenching, diuretic, anti-hypersensitive, hemostatic, and insecticidal properties [32]. Paulownia flowers are emerging as a new source for pharmaceuticals, foods, health products, cosmetics, and animal feed and many patents have been registered on these properties [10,33]. A study conducted on *P. tomentosa* flowers showed that they exhibited higher gallic acid, chlorogenic acid, quercetin, and luteolin than leaf extracts. The leaf extracts showed more catechin, caffeic acid, and coumaric acid than the flower extracts. In fact, catechins were found in larger amounts among the phenolic substances present in leaves and flower extracts [21]. The objectives of this study were to evaluate the effect of five drying methods (sun drying, shade drying, oven drying at 40, 60, 80, and 100 °C, freeze drying, and microwave drying) on leaves and flowers (pre- and post-anthesis stages) of *Paulownia elongata* and *P. fortunei* with respect to drying characteristics (drying time and drying rate) and retention of bioactive compounds (total polyphenols, flavonoids, catechins, tannins, and monomeric anthocyanins) for their use in health promoting products.

We conducted studies on the response of specific developmental stages of flowers (stage 6 and stage 9) that vary in pigmentation and size and juvenile leaf to five drying treatments. To the best of our knowledge, this is the first specifically designed study to investigate the suitability of a drying protocol to process biomass for the tea and quantify the total polyphenols, flavonoids, tannins, catechins, and resultant antioxidant capacities of these two *Paulownia* spp. It also takes forward our earlier research on the total polyphenol contents, flavonoids, and antioxidant capacity in mature leaves and their use as animal feed [33,34].

## 2. Materials and Methods

### 2.1. Plant Material and Sample Preparation

**Figure 1:**
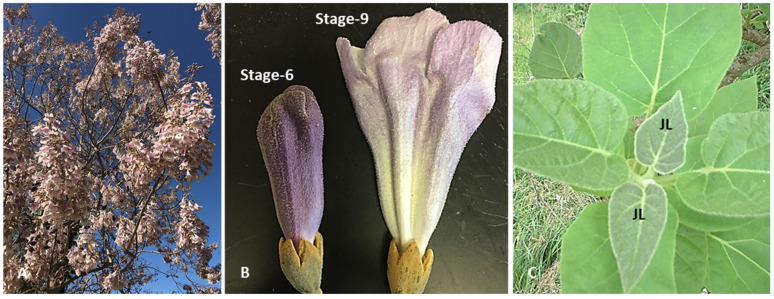
*Paulownia elongata* tree at the time of flowering and tissues used in the current study. (**A**) Paulownia tree with flowers, (**B**) flowers at stage 6 (pre-anthesis) and stage 9 (post-anthesis stage), and (**C**) Juvenile leaves (JL).

Flowering in *Paulownia* trees precedes leaf formation and lasts for 4–6 weeks (Figure 1A). *Paulownia elongata* and *P. fortunei* flowers (Figure 1B) and leaves (two fully expanded youngest ones; Figure 1C) were harvested from 8-year-old trees from the Fort Valley State University *Paulownia* demonstration plot. Plant material was gently dabbed with paper towel and calyx of the flower and leaf petioles were removed prior to drying treatments.

### 2.2. Drying Methods

Leaves and flowers (stages 6 and 9; Figure 1) of *Paulownia elongata* and *P. fortunei* were weighed in triplicates (50 g) and subjected to five drying treatments. Drying characteristics and total antioxidant potential (total polyphenols, flavonoids, catechins, tannins, monomeric anthocyanins, and antioxidant capacity) of dried *Paulownia* tissues were determined [13,35,36] and the values obtained for fresh tissues were used as control. Oven drying of flower and leaf tissues was conducted in a hot air oven (Heratherm^TM^ OGH60, Thermo Scientific, Inc., Waltham, MA, USA) at 40 (6 h), 60 (4 h), 80 (2 h), and 100 °C (1 and 24 h). The mass of the tissue was recorded every 20 min to determine the drying rate. Drying at 100 °C for 24 h was continued till equilibrium (no mass change) was reached. Sun drying of flower and leaf tissues was conducted at 27–30 ± 4 °C for 16 h at light intensity of 1200–1300 ± 500 µmolm^−2^s^−1^. The tissues were exposed to sunlight for 8 h per day for two days. The mass of the tissues was recorded every 4 h for 8 h to calculate the drying rate. Shade drying of the flowers and leaves was conducted at room temperature (25 ± 3 °C) for 1 week under natural airflow. Tissues were turned three times a day to improve aeration to avoid moisture pockets that might cause mold growth. The mass was recorded for drying-rate calculation and then left overnight at room temperature. For freeze drying, flowers and leaves were placed in Labconco Freezone Freeze Dryer System (Labconco Inc., Kansas City, MO, USA) containers at −20 °C for 24 h for pre-processing. The frozen flower and leave tissues were freeze dried at −55 °C and 0.300–0.400 ± 0.200 mBar vacuum for 48 h. Microwave drying was conducted using a GE 2.0 microwave oven (GE Appliances, Decatur, AL, USA) at 1200 W for 4 min 30 s. The drying rate was calculated as the amount of moisture removed per unit time per unit dry mass (g water g dry solid^−1^ min^−1^ m^−2^). Flowers and leaves were weighed (5 g in triplicates) and kept in a hot air oven at 103 °C for 24 h. The final mass of the tissues was recorded for all samples, and the moisture content and drying rates were analyzed.

### 2.3. Color Measurement

Fresh (control) and dried flowers and leaves were evaluated for their color using a HunterLab MiniScan XE plus (Hunter Associates Laboratory, Inc., Reston, VA, USA) colorimeter. The colorimeter was calibrated against a standard calibration plate of a white surface and set to CIE standard illuminant C [13]. The L*, a*, and b* values were determined and averaged from three readings taken at different places on the bed of the flowers and the leaves. Total color difference (ΔE) was calculated using the following equation [37]:

ΔE = ((ΔL*)^2^ + (Δa*)^2^ + (Δb*)^2^)^0.5^ (1)

where ΔL* = L* sample − L* control (difference in lightness and darkness) (positive = lighter; negative = darker)

Δa* = a* sample − a* control (difference in red and green) (positive = redder; negative = greener)

Δb* = b* sample − b* control (difference in yellow and green) (positive = yellow; negative = blue)

### 2.4. Water Activity Measurement

The water activity of dried and fresh flowers and leaves was measured at room temperature (25.4 ± 0.6 °C) using an AquaLab 4TE Dew Point water activity meter (METER Group, Pullman, WA, USA).

### 2.5. Methanolic Extract Preparation

Tissue samples were homogenized using liquid nitrogen (N_2_) and 5 g of homogenized powder was extracted with 35 mL HPLC grade 100% methanol. The flasks were left overnight (18 h) in dark on a temperature-controlled (28 °C) orbital shaker at 150 RPM. The suspension was centrifuged at 4000 RPM at 25 °C for 40 min and supernatant was collected. The tissue pellet was re-extracted with 15 mL methanol for 3 h and both extracts were combined, filtered, assayed, or stored at −80 °C in the dark.

### 2.6. Total Polyphenolic Content Estimation

Total polyphenolic content was determined by FC reagent method [38,39]. Nine different concentrations of gallic acid standards were pipetted (150 µL) followed by adding 600 µL of 7.5% sodium carbonate (*w*/*v*) and 750 µL of 2N FC reagent drop by drop. The ultraviolet/visible (UV/Vis) spectrophotometer Nanodrop 2000c (Thermo Fisher Scientific Inc., Waltham, MA, USA) was blanked using 2N FC reagent and absorption was read at 765 nm. The total phenolic content was calculated as gallic acid equivalents GAE/g of fresh and dry plant material based on a standard curve of gallic acid (*R*^2^ = 0.99). All determinations were carried out in triplicate.

### 2.7. Total Flavonoids Content Estimation

The aluminum chloride colorimetric method was used for total flavonoids estimation [39,40]. Various concentrations of quercetin dihydrate standard solutions and test solutions (0.5 mL) were mixed with 1.5 mL of 95% EtOH, 0.1 mL of 10% AlCl_3_, 0.1 mL of 1 M potassium acetate, and 2.8 mL of distilled water in 50 mL beakers. Solutions were then incubated at room temperature for 30 min and absorbance of the standard and test solutions was read using a spectrophotometer (Nanodrop 2000c) at 415 nm. Blank was prepared without the addition of AlCl_3_. Total flavonoids content was determined from the linear regression equation (R^2^ = 0.99) by the calibration curve of quercetin.

### 2.8. Total Tannins Assay

Total tannins content was determined by FC reagent method [38]. Tannic acid was used to prepare the standard curve.

### 2.9. Total Catechins Estimation (Diazotized Sulfanilamide Method)

Catechin-specific reagent (CSR) was prepared by dissolving 20 g sulfanilamide in 1000 mL double distilled warm water. Concentrated HCl (16 mL) was added, and the solution was kept on an ice bath to bring the temperature below 10 °C. The diazotization reaction was carried out by adding 20 mL of NaNO_2_. Precipitation of solid diazotized derivative of sulfanilamide started within 2 min as a pale-yellow lemon salt that was filtered using Whatman filter paper No. 2. The precipitate was dried under ice-cold conditions. For the catechin assay, 1% CSR (1% diazotized sulfanilamide) in absolute methanol (*w*/*v*) was prepared fresh. Ten different concentrations of catechin dihydrate solutions (25–900 mg/L) and test solutions were pipetted (50 µL) in cuvettes followed by adding 1 mL of CSR and 1 mL of 30% HCl (*v*/*v*). Reaction mixtures were incubated for 1 h at room temperature in the dark. The spectrophotometer was blanked using CSR and absorption was read at 425 nm using Nanodrop 2000c. Total catechin content was determined using the linear equation (R^2^ = 0.99) from the calibration curve of catechin dihydrate [41].

### 2.10. Total Monomeric Anthocyanin Measurement (pH Differential Method)

The pH differential method was used for total monomeric anthocyanin measurement [42,43]. Plant extract (500 µL) was pipetted into two disposable cuvettes. Then, 2 mL of buffers potassium chloride (KCl) (0.025 M; pH 1.0) and sodium acetate trihydrate (C_2_H_3_O_2_Na.3H_2_O) (0.4 M; pH 4.5) were added to the test sample (one buffer in each cuvette in a ratio of one part test sample to four-parts buffer). The solution was mixed and incubated for 30 min and absorbance was measured at 520 nm and 700 nm using distilled water as blank using Nanodrop 2000c spectrophotometer. The total anthocyanin content was calculated and expressed as Cyanidin-3-glucoside [43].

### 2.11. Antioxidant Capacity Measurement (TEAC Assay)

Total antioxidant capacity was measured using a 6-hydroxy-2,5,7,8-tetramethylchroman-2-carboxylic acid (TROLOX) equivalent antioxidant capacity assay [39,44]. A dilution series of 100–1200 µM TROLOX was prepared in 95% ethanol. The inhibition exerted by the standard TROLOX solution at the end of 6 min incubation determines the antioxidant capacity of the sample. The 2,2′-azino-bis (3-ethylbenzothiazoline-6-sulphonic acid) (ABTS) (38.4 mg) was dissolved in 10 mL of distilled water to make a 7 mM ABTS stock solution. A 7 mM ABTS solution was mixed with 6.6 mg of potassium persulfate (final conc. 2.45 mM) and incubated in dark at 25 °C for 16 h and diluted with absolute EtOH to obtain optical density (~0.70 ± 0.02) at 734 nm. This solution was used for the evaluation of plant extract. Diluted ABTS solution (2970 µL) and 30 µL of absolute ethanol were mixed (negative control) and plant extract (30 µL) was added to diluted ABTS solution (2970 µL), and the absorbance was read at 734 nm using Nanodrop 2000c after 6 min of the initial mixing and the same procedure was repeated in 30 s intervals for triplicates. Absolute ethanol was used as blank and measurements from extracts were plotted against TROLOX standards (calibration curve, R^2^ = 0.99) for percent inhibition at 6 min and expressed as TE µM/g. The following equation was used to calculate antioxidant capacity expressed as % inhibition:

% inhibition = [(Abs_control_ − Abs_sample_)/Abs_control_] × 100 (2)

where Abs_control_ = absorbance of control reaction (negative control); Abs_sample_ = absorbance of the test compound; and % inhibition = the inhibition of ABTS absorbance by TROLOX.

### 2.12. Statistical Analysis

Statistical analysis was carried out using the SAS^®^ system (version 9.4, SAS Institute, Cary, NC, USA). The analysis of variance (ANOVA) test was performed to evaluate whether there were significant effects of drying methods on the drying characteristics and total antioxidant capacity of *P. elongata* and *P. fortunei* [45]. When significant by ANOVA at *p* ≤ 0.05, the means were separated using the Tukey’s test.

## 3. Results and Discussion

### 3.1. Drying Curves and Drying Rate

The initial moisture content of *Paulownia* leaves and flowers was between 3.34–3.43 kg water/kg dry solid and 6.34–8.88 kg water/kg dry solid (dry basis, d.b.), respectively. Statistical analysis showed that the effects of the species (*P. elongata* and *P. fortunei*) and stages of flowers (stage 6 and stage 9) on the change in moisture contents with time and drying rates were not significantly different and, therefore, the data were pooled for the analysis.

#### 3.1.1. Drying Curves

**Figure 2:**
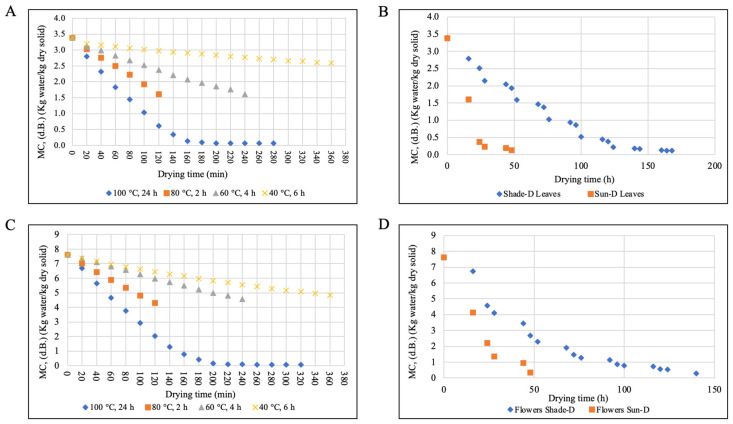
Changes in moisture content of Paulownia leaves and flowers during different drying methods. (**A**) Oven drying of leaves, (**B**) sun and shade drying of leaves, (**C**) oven drying of flowers, and (**D**) sun and shade drying of flowers.

Changes in the moisture contents of leaves and flowers during different drying methods are shown in Figure 2. Figure 2A,C illustrate changes in moisture content during oven drying at drying temperatures of 40 °C to 100 °C. The leaves reached 2.59 kg water/kg d.b. after 6 h of drying at 40 °C and reached the same moisture content after 31 min at 100 °C. The flowers reached 4.83 kg water/kg d.b. after 6 h of drying at 40 °C and reached the same moisture content after 44 min at 100 °C. From the curves (Figure 2A,C), it is observed that the drying temperature had a significant effect on the drying time. Figure 2B,D illustrate changes in moisture content during sun and shade drying. The leaves reached 1.6 kg water/kg d.b. after 16 h of sun drying and reached the same moisture content after 52 h of shade drying. The flowers reached the same moisture content after 25 h of sun drying and 69 h of shade drying. The drying time of shade drying was longer than that of sun drying. The equilibrium moisture contents (EMC) were reached at 200 and 240 min for leaves and flowers, respectively, at 100 °C (24 h). The EMCs were reached at 164 and 140 h for shade drying of leaves and flowers, respectively. For sun drying, the EMCs were reached at 48 h for both leaves and flowers.

#### 3.1.2. Drying Rates

**Figure 3:**
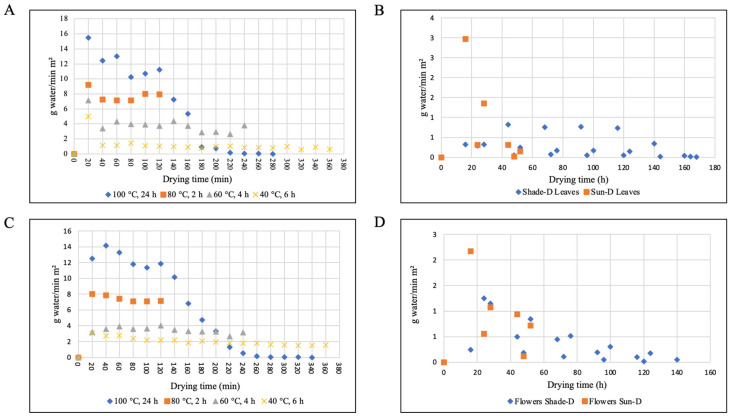
Changes in drying rate of Paulownia leaves and flowers during different drying methods (**A**) Oven drying of leaves, (**B**) sun and shade drying of leaves, (**C**) oven drying of flowers, and (**D**) sun and shade drying of flowers.

The drying rates of *Paulownia* leaves and flowers are shown in Figure 3. The average drying rates for *Paulownia* leaves were 6.26, 7.78, 3.90, and 1.17 g water g dry solid^−1^ min^−1^ m^−2^ for 100 °C (24 h), 80 °C (2 h), 60 °C (4 h), and 40 °C (6 h), respectively. The average drying rates for *Paulownia* flowers were 5.99, 7.44, 3.42, and 2.02 g water g dry solid^−1^ min^−1^ m^−2^ for 100 °C (24 h), 80 °C (2 h), 60 °C (4 h), and 40 °C (6 h), respectively. The drying rate was the highest at the first 1 h for all temperatures and decreased with time. The moisture contents of *Paulownia* leaves and flowers were very high during the initial phases of drying, which resulted in higher drying rates due to higher moisture diffusion. As the drying progressed, the loss of moisture in leaves and flowers caused a decrease in drying rates. The drying rate increased with an increase in temperature from 40 °C to 100 °C. The drying air temperature significantly affected (*p* < 0.5) the drying rates during preheat period (Figure 3A). It was higher in the first 20 min of drying, achieving 4.99 and 15.51 g water g dry solid^−1^ min^−1^ m^−2^ for a temperature of 40 and 100 °C, respectively. For flowers, it achieved 3.07 and 12.50 g water g dry solid^−1^ min^−1^ m^−2^ for a temperature of 40 and 100 °C, respectively (Figure 3C).

The time required to dry *Paulownia* leaves by sun and shade drying from an initial moisture content of 3.39 kg water/kg dry solid to a final moisture content of 0.13 kg water/kg dry solid was 48 and 160 h, respectively. The time required to dry *Paulownia* flowers by sun and shade drying from an initial moisture content of 7.63 kg water/kg dry solid to a final moisture content of 0.35 kg water/kg dry solid was 48 and 135 h, respectively. The average drying rates for *Paulownia* leaves were 0.27 and 0.85 g min^−1^ m^−2^ for shade and sun drying, respectively. The average drying rates for *Paulownia* flowers were 0.38 and 0.93 g min^−1^ m^−2^ for shade and sun drying, respectively.

### 3.2. Water Activity

The effects of the species (*P. elongata* and *P. fortunei*) and stages of flowers on the *a_w_* were not significantly different and, therefore, the data were pooled for the analysis. The water activity (*a_w_*) values of fresh flowers and oven-dried flowers at 40 °C (6 h), 60 °C (4 h), and 80 °C (2 h) varied from 0.960 to 0.985 and were not significantly different. The *a_w_* values (0.605–0.678) were not significantly different among sun-, shade-, and microwave-dried flowers. No significant difference was observed in *a_w_* values (0.166–0.239) between oven-dried flowers at 100 °C (24 h) and freeze-dried flowers. The *a_w_* values of fresh leaves and oven-dried leaves at 40 °C (6 h), 60 °C (4 h), and 80 °C (2 h) varied from 0.941 to 0.987 and were not significantly different. The *a_w_* values (0.248–0.277) were not significantly different among sun-, microwave-, and freeze-dried leaves. The *a_w_* values of oven-dried leaves at 100 °C (24 h) and shade drying were 0.347 and 0.425, respectively, and were significantly different (*p* < 0.05). Flowers and leaves that were oven dried at 100 °C (24 h), freeze-dried flowers, and sun-, microwave-, freeze-, and shade-dried leaves had *a_w_* values lower than 0.60; therefore, they were safe and stable with respect to microbial growth [46,47].

### 3.3. Color Analysis

The effects of the stages of flower and the effects of species on the leaf color parameters (L*—brightness, a*—greenness, and b*—yellowness) were not significantly different and, therefore, the data were pooled for the analysis. The effects of different methods of drying on the color parameters and overall color difference (∆E) of fresh and dried PE and PF flowers and leaves are presented in Table 1. A reduction in L* values was observed for dried flowers and leaves as compared to fresh flowers and leaves. The oven-dried flowers at 100 °C (24 h) and shade-dried leaves had the lowest values of L* compared to fresh samples. The reduction in L* values were because of concentration effects due to the loss of water during drying, surface deformation, and the generation of brown pigments [48].

The overall color difference (∆E) values increased significantly (*p* < 0.05) with an increase in drying air temperature for oven-dried flowers and leaves. The ∆E values were higher than 5, indicating that oven drying, freeze drying, microwave drying, and sun and shade drying resulted in perceived color changes by visual observation. Temperature and relative humidity during drying cause color degradation in dehydrated products [13].

### 3.4. Drying Treatments and Bioactive Compounds

At the completion of drying treatments, flower and leaf tissues of *P. elongata* and *P. fortunei* were processed for the estimation of the following compounds that are considered to contribute towards the total antioxidant capacity.

#### 3.4.1. Total Polyphenols

**Figure 4:**
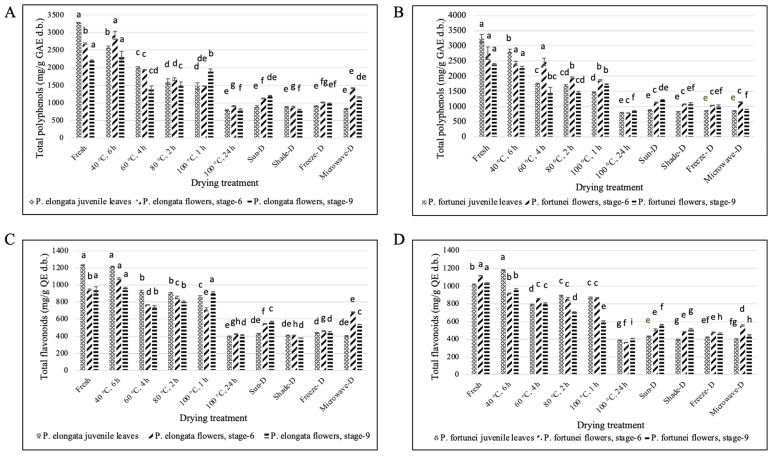
Effect of different drying treatments on the total polyphenol and total flavonoid contents of Paulownia tissues. (**A**) PE total polyphenol content, (**B**) PF total polyphenol content, (**C**) PE total flavonoid content, and (**D**) PF total flavonoid content. Same letters denote no statistically significant differences, *p* > 0.05.

The total phenolic contents of *Paulownia elongata* and *P. fortunei* fresh leaves were 3287.40 mg gallic acid equivalent (GAE) g^−1^ d.b. and 3243.03 mg GAE g^−1^ d.b., respectively. Drying treatments resulted in a significant decrease (*p* ≤ 0.05) in the total polyphenols in PE and PF leaves, with oven drying at 40 °C for 6 h causing the least reduction (2583.09 and 2826.32 mg GAE g^−1^ d.b., respectively), and oven drying at 100 °C for 24 h causing the largest reduction (793.20 and 804.99 mg GAE g^−1^ d.b., respectively). The recommended moisture content (3–5%) for the shelf life of tea was achieved by oven drying at 100 °C for 24 h, sun drying, shade drying, freeze drying, and microwave drying. Among these five treatments, sun drying was the best treatment for PE leaves (893.50 mg GAE g^−1^ d.b.) and freeze drying was the best treatment for PF leaves (857.69 mg GAE g^−1^ d.b.) for total polyphenols retention (Figure 4A,B).

The total phenol analyses of the fresh flowers (stage 6) of PE and PF showed 2684.61 mg GAE g^−1^ d.b. and 2751.05 mg GAE g^−1^ d.b., respectively. The fresh flowers of PE and PF (stage 9) exhibited 2203.40 mg GAE g^−1^ d.b. and 2378.35 mg GAE g^−1^ d.b. total polyphenols, respectively. After drying treatments, a significant decrease (*p* ≤ 0.05) in the total polyphenols was observed in both the stages of flowers (stages 6 and 9), except for oven-drying at 40 °C for 6 h. In fact, PE flower stage 6 showed a significant increase in total polyphenols at oven drying at 40 °C for 6 h (2917.79 mg GAE g^−1^ d.b.). At optimum moisture content (3–5%), the best treatment for the extended shelf life of stage 6 flowers of PE and PF was microwave drying (1152.98 and 1144.16 mg GAE g^−1^ d.b., respectively), whereas for stage 9 flowers, sun drying (1201.88 and 1229.84 mg GAE g^−1^ d.b., respectively) was the best drying treatment (Figure 4A,B).

#### 3.4.2. Total Flavonoids

The total flavonoid content of PE and PF tissues showed similar patterns as those of total polyphenols. The total flavonoid contents showed by the fresh leaves of PE and PF were 1231.32 and 1019.51 mg quercetin equivalent (QE) g^−1^ d.b., respectively. After various drying treatments, a significant decrease (*p* ≤ 0.05) in the total flavonoids of PE and PF leaves was observed, except for oven drying at 40 °C for 6 h (1072.60 and 1180.05 mg QE g^−1^ d.b., respectively). At optimum moisture content (3–5%), freeze drying was the best treatment for PE leaves (438.09 mg QE g^−1^ d.b.), and sun drying was the best treatment for PF leaves (507.05 mg QE g^−1^ d.b.) (Figure 4C,D).

The total flavonoid contents of fresh stage 6 flowers of PE and PF were 945.26 and 1116.05 mg QE g^−1^ d.b. for PE and PF, respectively. Stage 9 fresh flowers recorded 937.02 and 1032.96 mg QE g^−1^ d.b. total flavonoids, respectively. After drying treatments, the total flavonoid contents of PE and PF flowers showed the same pattern as that of total polyphenols. There was a significant decrement in the total flavonoids after drying treatments, except for PE flowers (stage 6) dried at 40 °C for 6 h. At optimum moisture content (3–5%), microwave drying was the best for PE and PF flowers (stage 6) (685.86 and 554.35 mg QE g^−1^ d.b., respectively), whereas sun drying was the best for stage 9 (575.49 and 563.33 mg QE g^−1^ d.b., respectively) (Figure 4C,D).

#### 3.4.3. Total Tannins and Catechins

**Figure 5:**
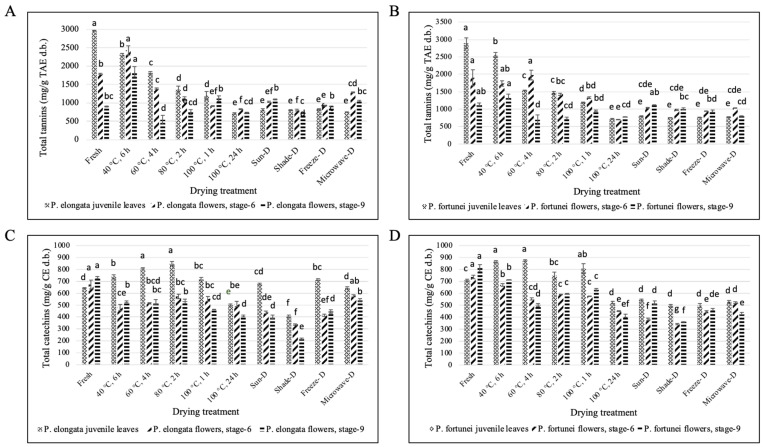
Effect of different drying treatments on the total tannin and total catechin contents of Paulownia tissues. (**A**) PE total tannin content, (**B**) PF total tannin content, (**C**) PE total catechin content, and (**D**) PF total catechin content. Same letters denote no statistically significant differences, *p* > 0.05.

The leaves of PE and PF showed similar patterns in total tannins. Total tannins for fresh leaves for PE and PF were 2958.37 and 2894.97 mg TAE g^−1^ d.b., respectively. After each drying treatment, the tannins started decreasing significantly (*p* ≤ 0.05). Oven-dried leaves (at 40, 60, 80, and 100 °C for 6, 4, 2, and 1 h, respectively) showed significantly higher values for total tannins than oven drying at 100 °C for 24 h and sun, shade, freeze, and microwave drying, which is mainly due to the difference in their moisture content. At optimum moisture content (3–5%), the best treatment for PE leaves was freeze drying (822.48 mg TAE g^−1^ d.b.), whereas for PF leaves, it was the sun drying (799.18 mg TAE g^−1^ d.b.); however, there was no significant difference among oven drying at 100 °C for 24 h and sun, shade, freeze, and microwave drying in both PE and PF leaves (Figure 5A,B).

The total tannins for PE and PF leaves were significantly reduced after the drying treatments. At optimum moisture content, there was no significant difference among oven drying at 100 °C for 24 h and sun, shade, freeze, and microwave drying for PE and PF leaves (*p* > 0.05). Total tannins for flowers (stages 6 and 9) were in accordance with the results obtained for total polyphenols and flavonoids. *Paulownia elongata* flowers (stages 6 and 9) showed higher tannins after oven drying at 40 °C for 6 h and the levels of total tannins dropped in subsequent oven-drying treatments. The best treatment at optimum moisture content for PE and PF flowers (stage 6) was microwave drying (1046.69 mg TAE g^−1^ d.b.) and sun drying (1035.70 mg TAE g^−1^ d.b.), respectively. However, there was no significant difference among sun, shade, and microwave drying for stage-6 PF flowers. Oven drying at 40 °C for 6 h showed the highest total tannins for PE and PF flowers (stage 9); however, at optimum moisture content, the best treatment was sun drying for PE and PF flowers (stage 9) (1088.16 and 1114.37 mg TAE g^−1^ d.b., respectively) (Figure 5A,B).

#### 3.4.4. Total Catechins and Monomeric Anthocyanins Content

Unlike total tannins, catechins increased after various drying treatments for PE and PF leaves. The best treatment at optimum moisture content for PE and PF leaves were freeze- (715.57 mg CE g^−1^ d.b.) and sun drying (542.29 mg TAE g^−1^ d.b.), respectively. However, there was no significant difference among oven drying at 100 °C for 24 h and sun, shade, freeze, and microwave drying for PF leaves. Unlike leaves, the total catechins for PE and PF flowers (stages 6 and 9) reduced significantly (*p* ≤ 0.05) after the drying treatments. The best treatment for PE flowers (stages 6 and 9) at optimum moisture content was microwave drying (579.70 and 540.90 mg CE g^−1^ d.b., respectively). For PF flowers (stage 6), the best treatment was microwave-drying (521.61 mg CE g^−1^ d.b.) and sun drying was the best for stage 9 (521.69 mg CE g^−1^ d.b.) (Figure 5C,D).

**Figure 6:**
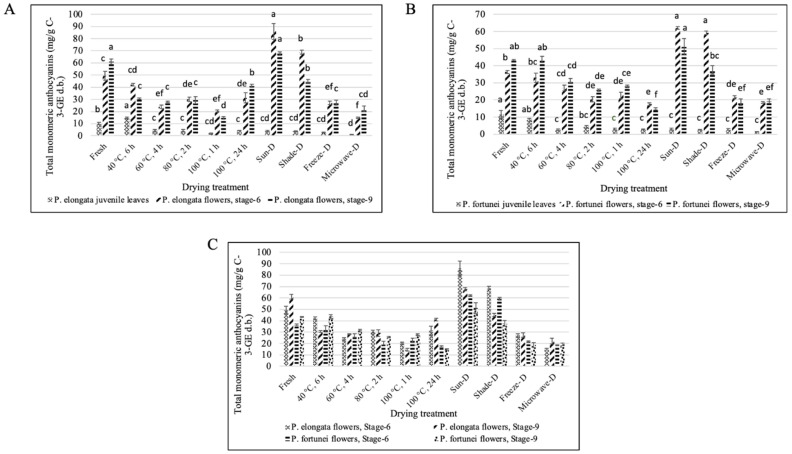
Effect of different drying treatments on the total monomeric anthocyanins of Paulownia tissues. (**A**) PE total monomeric anthocyanins content, (**B**) PF total monomeric anthocyanins content, and (**C**) comparison of monomeric anthocyanins between *P. elongata* and *P. fortunei* flowers (stages 6 and 9). Same letters denote no statistically significant differences, *p* > 0.05.

The fresh leaves of PE and PF showed very low anthocyanin content that further reduced after drying treatments showing the lowest amount with microwave drying for both PE and PF (0.81 and 1.42 mg C-3-GE g^−1^ d.b., respectively). Total monomeric anthocyanins were found higher in PE flowers than PF flowers. Both PE and PF flowers at stage 6 showed significantly higher anthocyanins than stage 9 (*p* ≤ 0.05). The best treatment for PE and PF flowers (stages 6 and 9) was sun drying followed by shade drying at optimum moisture content. *Paulownia elongata* flowers (stage 6) registered the highest amount of total monomeric anthocyanins (85.71 mg C-3-GE g^−1^ d.b.) among all four types of flowers (Figure 6A–C).

#### 3.4.5. Total Antioxidant Capacity (TAC)

The total antioxidant capacity was significantly reduced after various drying treatments for all the tissues of PE and PF. The PE and PF fresh flowers (stage 6) registered the highest antioxidant capacity in comparison with the other tissues (13,671.97 and 11,894.85 µM TE d.b.) (Table 2). At optimum moisture content, the antioxidant capacity was reduced significantly in PE and PF tissues (*p* ≤ 0.05). The total antioxidant capacity is in accordance with the total polyphenolic content, which shows a strong correlation between total polyphenols and the total antioxidant capacity. High TPP is generally associated with high TEAC and vice versa. This agrees with previous studies, which show a linear relationship between TPC and total antioxidant capacities (TAC) [49].

Oven drying at 100 °C for 24 h and sun, shade, freeze, and microwave drying showed low antioxidant capacity, most probably due to the enzymatic degradation of antioxidant compounds in two *Vitex* species [35]. Longer drying treatments at high temperature (100 °C for 24 h) were highly detrimental as the antioxidant potential significantly decreased. Significant differences in antioxidant levels (polyphenols, flavonoids, and tannins) were observed among the fresh, oven-dried (40, 60, 80, and 100 °C), and sun-, shade-, freeze-, and microwave-dried leaves and flowers of PE and PF. This study showed that drying plant materials at low temperatures for a longer duration (sun, shade, and freeze drying) or at a high temperature for a longer duration (100 °C for 24 h) significantly decreased the antioxidants. Prolonged and intense thermal treatment can cause significant loss because of the heat instability of compounds [50]. Oven drying at 100 °C for 1 h does not cause a significant loss in antioxidants, which suggests that the duration of drying is one of the critical factors that is directly correlated with the moisture content levels of the plant material. In this study, we found that oven drying at a low temperature (40 °C for 6 h) increased the total polyphenols, total flavonoids, and total tannins in PE flowers (stages 6 and 9). Yi and Wetzstein, 2011 [51] reported that drying at a low temperature can increase the total polyphenol contents and antioxidant capacity while studying herb species from Lamiaceae. Sun drying and 40 °C oven drying resulted in significantly higher TPC and TEAC than fresh samples of peppermint and motherwort, while oven drying at 70 °C negatively affected the TPC and TEAC. Air drying of oregano and peppermint at 25 °C to 32 °C resulted in significant increases in TPC [52]. During drying, metabolically active plants slowly lose their moisture and might sense the moisture loss as stress, leading to the production of more antioxidants as a defense mechanism. It is reported that the synthesis of several phenylpropanoid compounds (flavonoids, is flavonoids, psoralens, coumarins, phenolic acids, lignin, and suberin) was induced in plants in response to biotic and abiotic stress, such as wounding, low or high temperature, and pathogens’ attack [53]. An increase in safrole, myristicin, and other bioactive compounds with elevated free radical scavenging activity was observed because of heating (180 °C) in nutmeg (*Myristica fragrans*) essential oil [50]. Also, moisture content is an important factor for the final antioxidant capacity. Drying at 40 °C (oven drying) for 6 h increased the antioxidant capacity, which could be the result of the availability of optimum moisture content required to keep the antioxidants intact. When the tissue was dried at a low temperature (25–28 °C) during shade drying for about a week, it lost 70–90% of its water, which resulted in a loss in antioxidant levels and capacity. Table 2 shows the % inhibition of free radical (ABTS^+^) by PE and PF tissues before and after drying treatments. Oven drying either for a shorter duration or at a low temperature showed higher antioxidant capacity, whereas oven drying for a longer duration and high temperature (100 °C for 24 h) significantly reduced the antioxidant capacity, showing the least % inhibition in PE and PF (Table 2). The possible reason for this could be that the metabolically active tissues become damaged by direct heat due to exposure and degrade significantly, which leads to loss in their activity. Hence, this study reveals that moisture content plays a vital role in the antioxidant potential of plants. Studies conducted on tea leaves suggest that the enzymatic reactions of biosynthetic pathways in their early phase are terminated due to heat and moisture loss during drying and new compounds are formed due to degradation [12]. Similarly, cell wall and membrane degradation may be linked with drying and may play an important role in decreased antioxidant level and activity after drying the plant material [51].

The total monomeric anthocyanins increased significantly when subjected to sun and shade drying. In *Camellia sinensis* (green tea), oven, microwave, sun, shade, and freeze drying led to significant increase in total polyphenolic (TPC) and total flavonoid content (TFC), with oven drying at 60 °C and 100 °C causing the highest increase in TPC and TFC, respectively [13]. The pattern of our results was similar to these results. It could be because the drying process can break down the cellular constituents, leading to the accelerated release of anthocyanins from the food matrix [54,55]. Another reason could be the presence of active enzymes in the fresh plant material that thwarts the release of these bioactive compounds, whereas dried samples have a lower water content that inactivates the enzymes causing high levels of phenolic compounds to remain in the extract [56]. Polyphenol oxidase, an enzyme responsible for the oxidation of phenolic compounds into highly reactive quinones, is completely inactivated at 80 °C for 10 min [57]. The enhanced anthocyanins could be associated with the inactivation of deteriorative enzymes such as lipoxygenase and polyphenol oxidases, which oxidize diphenols in the presence of oxygen, which leads to the enzymatic oxidation of antioxidants in medicinal plants [51,57,58]. Drying may cause a faster and higher release of antioxidants from the cells due to the breakdown of cellular constituents, which is in agreement with previous studies [59,60,61]. Drying at a high temperature for a longer duration can enhance the yield of various antioxidants including anthocyanins due to the degradation of cell membrane and destruction of cell wall [51]. Also, the monomeric anthocyanins might tend to change their form during sun- and shade-drying processes, which could be the reason for the intensification of these compounds.

## 4. Conclusions

*Paulownia* flowers and leaves are suitable for developing a herbal tea with medicinal compounds. Drying is one of the most critical unit operations that makes tea shelf-stable and retains most of the beneficial bioactive compounds, preserves the original color as much as possible, and does not degrade the taste. The higher moisture content in *Paulownia* tissues results in a higher concentration of total polyphenols and flavonoids. This translates into a higher total antioxidant capacity in fresh tissues. A strong correlation was observed between the moisture content and total polyphenols (the lower the moisture content, the lower the polyphenols). Oven drying at 40, 60, 80, and 100 °C for 6, 4, 2, and 1 h, respectively, exhibits higher antioxidant activity than oven drying at 100 °C for 24 h in the leaves and flowers of *P. elongata* and *P. fortunei*. This could be attributed to decreased free radical scavenging activity resulting from heating for a prolonged time, which leads to lower moisture content. A high temperature for a longer duration (oven drying at 100 °C for 24 h) adversely affects the bioactive compounds. Although our results indicate that drying results in a significant loss in the total antioxidants and their activity, it is necessary to determine the best treatment that results in the optimum moisture content for the shelf life of the tea. Hence, it is important to find a drying treatment for *Paulownia* tea that retains the antioxidant capacity, color, and other organoleptic properties as much as possible while having the optimum moisture content (3–5%) for shelf life. The current study indicates that sun and shade drying are the best drying methods for commercially producing *Paulownia* tea based on the overall effect on the antioxidants levels, antioxidant capacity, moisture content (10–36% wet basis with respect to the total initial weight of the material), and drying rate. The moisture content could be further reduced by prolonging the duration of drying at the commercial level. Sun drying leads to a considerable reduction in the drying time in comparison to shade drying while retaining the product quality as much as possible (i.e., color, nutrient/antioxidant retention). It is useful to quantify the bioactive phytochemicals present in a plant to estimate the potential health benefits and possible applications. *Paulownia* leaves and flowers at stage 6 are the better tissues for total antioxidant retention. *Paulownia* has a much lower amount of catechins in comparison to *Camellia sinensis*; however, the high overall polyphenol level in *Paulownia* leaves may contribute to health benefits, making a strong case for a herbal tea. The study also indicates the possibility of developing teas by mixing leaf and flower tissues to balance color, flavor, taste, and medicinal properties. *Paulownia* tissues can also be mixed with already established teas in the market. *Paulownia* tissues present a potential case for developing various kinds of teas for an increasing market; however, future research needs to define the actual magnitude of different types of antioxidants their health benefits, establish the safe range of consumption associated with these benefits, and elucidate potential mechanisms of action.

## Figures and Tables

**Table 1:** Color characteristics of *P. elongata* and *P. fortunei* flowers and leaves in response to various drying treatments.

Type of Drying	Flowers								Leaves			
	*Paulownia elongata*				*Paulownia fortunei*							
	L	a	b	∆ E	L	a	b	∆ E	L	a	b	∆ E
Fresh	52.33	8.55	−3.22		70.24	4.56	8.12		42.87	−6.88	20.88	
40 °C, 6 h	47.69	8.58	−2.33	10.43 ^e^	66.28	5.47	10.36	12.84 ^e^	41.39	−6.67	20.90	6.74 ^e^
60 °C, 4 h	46.51	9.88	17.67	22.84 ^c,d^	50.69	8.98	21.73	25.58 ^c,d^	36.27	1.95	15.05	13.57 ^c^
80 °C, 2 h	42.29	10.99	21.19	27.91 ^b^	47.00	11.21	25.14	30.14 ^b,c^	36.77	2.25	14.38	13.6 ^c^
100 °C, 1 h	39.45	10.54	18.39	25.87 ^b,c^	44.92	11.58	23.09	30.59 ^b,c^	35.53	1.59	12.96	15.05 ^b,c^
100 °C, 24 h	30.62	10.69	20.09	33.09 ^a^	29.51	10.03	17.40	42.28 ^a^	31.51	3.44	12.92	18.52 ^a,b^
Freeze	38.29	3.56	9.59	22.72 ^c,d^	40.44	10.87	17.39	32.58 ^b^	38.90	−5.42	17.03	8.71 ^d,e^
Microwave	39.61	5.20	16.86	26.22 ^b,c^	44.35	6.82	21.01	31.33 ^b,c^	35.93	−1.65	16.66	11.99 ^c,d^
Sun	41.31	4.44	8.89	18.7 ^d^	50.43	2.14	14.75	23.61 ^d^	30.57	0.13	12.39	17.31 ^a,b^
Shade	37.92	3.71	11.82	23.03 ^c,d^	45.47	5.75	16.95	27.03 ^b,c,d^	28.84	0.66	10.03	20.26 ^a^

Same letters denote no statistically significant differences, *p* > 0.05. ∆ E = Overall color difference

**Table 2:** Total antioxidant capacity of fresh and dried leaf and flower tissues of *P. elongata* and *P. fortunei*.

Antioxidant Capacity						
µM TROLOX Equivalent/g of Fresh/Dry Matter						
Drying Treatment	*P. elongata* Leaves	*P. fortunei* Leaves	*P. elongata* Flowers, Stage 6	*P. fortunei* Flowers, Stage 6	*P. elongata* Flowers, Stage 9	*P. fortunei* Flowers, Stage 9
Fresh	7692.7 ± 11.9	8076.4 ± 14.0	13,672.0 ± 10.1	11,894.9 ± 76.4	6270.5 ± 336.5	6447.3 ± 98.9
40 °C, 6 h	6056.8 ±8.4	6594.4 ±7.0	9181.6 ± 27.4	10,906.9 ± 15.6	8066.2 ± 39.2	9994.2 ± 145.6
60 °C, 4 h	4408.9 ± 19.9	4682.6 ± 13.3	7430.8 ± 63.0	8461.0 ± 46.8	4584.2 ± 169.1	7319.9 ± 129.7
80 °C, 2 h	3637.1 ± 63.1	2517.0± 68.3	3944.9 ± 171.3	7087.4 ± 89.5	3213.7 ± 7.4	4509.8 ±78.7
100 °C, 1 h	2329.6 ± 80.8	1794.3 ± 102.0	2589.8 ± 263.9	5566.2 ± 63.6	2604.9 ± 79.6	2767.1 ± 201.4
100 °C, 24 h	1787.7 ± 9.0	1520.1± 24.0	2010.5 ± 3.8	1753.9 ± 7.1	1900.9 ± 9.1	1999.5 ± 10.1
Sun-D	2058.1 ±5.6	2062.4 ± 2.8	2581.2 ± 6.7	2547.0 ± 3.6	2782.9 ±4.2	2825.28 ± 9.0
Shade-D	2035.8 ±4.7	2007.8 ±2.8	1979.4 ± 5.2	2410.2 ± 4.2	1745.0 ±5.0	2501.8 ± 6.8
Freeze-D	2067.9 ±3.1	2119.7 ± 2.2	2094.5 ± 10.0	2339.1 ± 7.5	2208.0 ± 13.8	2273.4 ± 4.1
Microwave-D	1916.4 ± 10.8	1944.5 ± 1.4	3215.9 ± 15.2	2697.7 ± 6.8	2536.7 ± 12.9	2160.4 ± 6.2

(Mean ± SE).

## Data Availability

Data contained within the article and available upon request.
